# A comparative analysis of sleep spindle characteristics of sleep-disordered patients and normal subjects

**DOI:** 10.3389/fnins.2023.1110320

**Published:** 2023-03-30

**Authors:** Chao Chen, Kun Wang, Abdelkader Nasreddine Belkacem, Lin Lu, Weibo Yi, Jun Liang, Zhaoyang Huang, Dong Ming

**Affiliations:** ^1^Academy of Medical Engineering and Translational Medicine, Tianjin University, Tianjin, China; ^2^Key Laboratory of Complex System Control Theory and Application, Tianjin University of Technology, Tianjin, China; ^3^Department of Computer and Network Engineering, College of Information Technology, United Arab Emirates University, Al Ain, United Arab Emirates; ^4^Zhonghuan Information College Tianjin University of Technology, Tianjin, China; ^5^Beijing Machine and Equipment Institute, Beijing, China; ^6^Department of Rehabilitation, Tianjin Medical University General Hospital, Tianjin, China; ^7^Department of Neurology, Xuanwu Hospital, Capital Medical University, Beijing, China; ^8^Beijing Key Laboratory of Neuromodulation, Beijing, China

**Keywords:** sleep spindles, EEG, sleep disorders, fusion algorithm, sleep spindle characteristics

## Abstract

Spindles differ in density, amplitude, and frequency, and these variations reflect different physiological processes. Sleep disorders are characterized by difficulty in falling asleep and maintaining sleep. In this study, we proposed a new spindle wave detection algorithm, which was more effective compared with traditional detection algorithms such as wavelet algorithm. Besides, we recorded EEG data from 20 subjects with sleep disorders and 10 normal subjects, and then we compared the spindle characteristics of sleep-disordered subjects and normal subjects (those without any sleep disorder) to assess the spindle activity during human sleep. Specifically, we scored 30 subjects on the Pittsburgh Sleep Quality Index and then analyzed the association between their sleep quality scores and spindle characteristics, reflecting the effect of sleep disorders on spindle characteristics. We found a significant correlation between the sleep quality score and spindle density (*p* = 1.84 × 10^−8^, *p*-value <0.05 was considered statistically significant.). We, therefore, concluded that the higher the spindle density, the better the sleep quality. The correlation analysis between the sleep quality score and mean frequency of spindles yielded a *p*-value of 0.667, suggesting that the spindle frequency and sleep quality score were not significantly correlated. The *p*-value between the sleep quality score and spindle amplitude was 1.33 × 10^−4^, indicating that the mean amplitude of the spindle decreases as the score increases, and the mean spindle amplitude is generally slightly higher in the normal population than in the sleep-disordered population. The normal and sleep-disordered groups did not show obvious differences in the number of spindles between symmetric channels C3/C4 and F3/F4. The difference in the density and amplitude of the spindles proposed in this paper can be a reference characteristic for the diagnosis of sleep disorders and provide valuable objective evidence for clinical diagnosis. In summary, our proposed detection method can effectively improve the accuracy of sleep spindle wave detection with stable performance. Meanwhile, our study shows that the spindle density, frequency and amplitude are different between the sleep-disordered and normal populations.

## 1. Introduction

The American Academy of Sleep Medicine (AASM) scoring manual defines sleep spindles as a series of waveforms seen on electroencephalographic (EEG) leads during polysomnography, with a frequency from 11 to 16 Hz and a duration of more than 0.5 s. Generally, in the case of limited channels, spindles are divided into slow spindles (11–13 Hz), typically found in the frontal derivations, and fast spindles (13–16 Hz), usually seen with maximal amplitude over the central derivations (Iber et al., 2007). The sleep spindle wave represents the exchange of information between the thalamus and the cerebral cortex, and it lasts for 0.5 s or longer (usually 0.5–1.5 s). These waveforms are generated in the thalamic reticular nucleus and project to cortical neurons (Lotchev et al., 2019), besides, there is substantial evidence now that their generation may also happen locally in the cortex, where the EEG signals are measured.

One of the chief roles of spindles is to protect the sleeping brain from external sensory stimuli and function as a biomarker of sleep integrity (Dang-Vu et al., 2010; Saletin and Walker, 2013; Thanh et al., 2015). Sleep spindles are vital in the analysis of intellectual functioning during sleep. As different spindle features may represent specific features of cortical development, we can identify human development by examining spindle density, frequency, and power, as well as the percentage of fast spindles. Spindle density may reflect thalamocortical coherence or connectivity, spindle frequency polarization of thalamocortical neurons (Goldstone et al., 2019), spindle power white matter integrity around the thalamus (Fernandez and Lüthi, 2020), and fast spindle hippocampal development (Saletin et al., 2013). Therefore, examining these indicators of sleep spindles is essential to understand neural development. Changes in the characteristics of sleep spindles are observed in several diseases, such as Parkinson’s disease (Christensen et al., 2014; Latreille et al., 2015), schizophrenia (Wamsley et al., 2012), autism, insomnia, and Alzheimer’s disease, and these changes may represent biomarkers of diseases and have critical complementary diagnostic value. Therefore, it is imperative to detect spindles accurately.

A number of studies have attempted to identify the best independent aspects of spindle activity, including, among others, density, duration, amplitude, and frequency. Studies have also tried to ascertain if it matters when the spindles occur: earlier or later, or in the course of life. Hahn et al. (2019) observed a longitudinal increase in spindle density and frequency, which seems to be driven by fast spindles (13–15 Hz) during the transition period from 8–11 years to 14–18 years (*n* = 34). Goldstein et al. (Michael et al., 2021) completed overnight polysomnographic recording and subsequent daytime tests in the laboratory of 15 insomniacs and 15 well-sleeping controls, showing a trend toward increased sleep spindles density. DelRosso et al. (2021) recorded and analyzed polysomnographic data of children with restless sleep disorder (RSD) and of normal controls. They found that the duration of frontal spindles in stage N2 of sleep was longer in children with RSD than in controls, and the frontal spindle density and intensity tended to increase in children with RSD. They found no significant differences in the central spindles. Researchers have documented that spindles begin to develop as early as 1 month of age; however, the rate of development of spindles varies throughout the lifespan (Hoedlmoser et al., 2014). The duration, amplitude, and individual properties of the spindle vary with age (Goldstone et al., 2019).

Altogether, spindle activity may be considered a suitable marker of brain development (Gruber and Wise, 2016). In this paper, through the study of various conventional sleep spindle wave automatic detection algorithms, we innovatively propose a multi-algorithm fusion spindle wave automatic detection algorithm to optimize the performance of spindle wave automatic detection, using Morlet wavelet algorithm, RMS algorithm and improved k-means clustering algorithm, and the improved spindle wave detection algorithm is able to perform equally well and consistently in two different datasets: sleep-disordered patients and normal people. Besides, we analyzed the density, amplitude, and frequency characteristics of the spindles of the C3 channel of the central brain region and the difference analysis of the number of spindles between two groups of symmetrical channels, C3/C4 and F3/F4, based on the differences between the sleep-disordered subjects and normal subjects (those without any sleep disorder), primarily from the perspective of the sample as a whole as well as the individual. We found that spindle density constantly decreased as the sleep quality score increased (the higher the score, the worse the sleep quality), i.e., the higher the spindle density, the better the sleep quality. We found no correlation between sleep quality and frequency in the overall distribution of the two categories of subjects, namely, sleep-disordered and normal. The average amplitude of the spindle decreased as the score increased, and the average amplitude of the spindle of the sleep-disordered subjects was lower. The number of spindles was almost the same between the symmetric channels, with no variability.

## 2. Materials and methods

The data collection for this experiment came from the Department of Neurology of Xuanwu Hospital in Beijing, China. We analyzed the data of 30 subjects (20 sleep-disordered and 10 normal subjects). The subjects in this experiment were between 20–40 years old to avoid, as far as possible, the influence of differences in the density and frequency of the spindles between people of different ages on the experimental results. In this experiment, sleep disorder subjects were selected from people with insomnia disorder and sleep apnea disorder. When the 20 sleep disorders were selected, an initial screening had been performed to ensure that they had the same symptoms. A Pittsburgh Sleep Quality Index (PSQI) sleep quality assessment was also performed prior to EEG signal acquisition, and the subjects’ sleep disorders were also assessed by a physician based on a combination of relevant medical history and medication use to ensure that they had the disease. The 10 subjects in the normal control group were healthy individuals. All 30 subjects were recruited from the society.

The experiment used a polysomnography (PSG) to collect 32 channels of EEG data from subjects, with bilateral mastoid as the average reference electrode, and the electrode impedance was set to below 25 kΩ. At the same time, hospitals use PSG to collect ECG data, EMG data, eye movement data, and respiratory tension data, etc. The collection and analysis of these physiological signals can help physicians to diagnose sleep-related diseases and determine their severity. Since this study is based on EEG signals, only the collected EEG signals are analyzed in this paper. To ensure the accuracy of the physiological signal collection, the subjects were required to prohibit the intake of alcohol and the consumption of caffeine, sedatives, hypnotics, and other relevant drugs that could affect data collection 1 week before the sleep monitoring. Prior to data collection, we arranged for the subjects to take a bath and clean their heads to ensure that the electrodes were in good contact with the skin to facilitate data collection. In addition, patients were informed to urinate and defecate in advance to avoid getting out of bed at night, which could have caused unnecessary effects on data collection, or they could keep a disposable night pot alongside their bed. Next, the experimenter recorded the basic information of the patient such as name, weight, gender, age, etc. When the electrode connection was carried out, the relevant electrode connection points were wiped with special cleaning cream for electrode cleaning to ensure accurate data collection, and the electrode placement ensured the accuracy of the electrode position and the firmness of placement and be placed in the prescribed order. After the pre-acquisition work was prepared, the data collection software was turned on to collect data. During the data acquisition process, turn off the cell phone to avoid interference with the experimental data from the external environmental sound. When the acquisition was completed in the morning, the device was turned off, and the patient was woken up. At this point, the experimental data collection was completed. Subjects entered the monitoring room by 7:30 p.m. to familiarize themselves with the environment and began recording data at 9:00 p.m., with each data length starting at 9:00 p.m. and ending at 6:30 a.m. Since the sleep time of each subject was not uniform, in order to analyze the differences in the characteristics of individual and overall sleep spindle waves, the EEG data of up to 8 h from the first sleep stage was intercepted for the study according to the stages of sleep staging.

With the deepening of spindle research, a large number of spindle detection algorithms have been proposed, such as Morlet wavelet detection algorithm, small-window root mean square (RMS) detection algorithm, and hidden Markov model–support vector machine (HMM–SVM) detection algorithm. However, these algorithms do not perform well in some performance metrics. Athanasios and Clifford (2015) proposed a probabilistic wavelet estimation algorithm based on wavelet algorithm for automatic detection of spindle waves, and although this algorithm has a more desirable accuracy, the performance in recall does not improve significantly, reaching only about 70% on average. The performance of small-window RMS algorithm in precision is relatively general. Although the HMM–SVM algorithm uses the fusion of HMM and SVM algorithms and offers some improvements in the mean value of evaluation metrics, but its stability in a large number of samples is not ideal, and the operation process of the method based on two classification models is very complicated. In addition to the shortcomings in each evaluation index, at present, there is also a shortage of public databases for spindle detection, and the only well-known databases are the MASS database and the DREAMS database. The lack of databases has led to difficulties in validating the stability of different detection algorithms. Based on the above shortcomings of spindle detection algorithms, we propose an algorithm based on the fusion of Morlet wavelet, small-window RMS, and improved K-means for spindle detection. The experimental results showed that this fusion algorithm can effectively improve the performance of spindle detection and showed a certain stability in different samples.

The specific steps of the fusion algorithm proposed in this paper are as follows: (1) The pre-processed sleep EEG signal is fed into two spindle wave automatic detectors, Morlet wavelet and small-window RMS, and the spindle wave output from each detector is then discriminated as true or false. (2) The spindle wave result sets of the two detectors are counted, and the detected identical spindles are grouped into the set of overlapping spindles. In this study, when two spindles overlap in the time series, this paper considers these two spindle waves as overlapping spindle waves, and the spindle wave with longer duration is selected as the sample in the set of overlapping spindles. (3) The set of non-coincident spindle waves is put into the improved k-means clustering algorithm. In this paper, the amplitude and frequency of non-coincident spindles are extracted as variables for k-means clustering, because they are the main features of spindles, and the differences of spindles after automatic detection are mainly reflected in the amplitude and frequency, after which the clusters with a large number of non-spindle waves after clustering are discarded in this paper, leaving the clusters of true spindle waves. (4) The clustered spindle wave set and the overlapping spindle wave set are combined as the final result set of this paper.

In this paper, the true spindle waves hand-picked by experts are defined as the two-two intersection of three experts’ detection results or the intersection of three. The non-intersecting part of the spindle waves picked by three experts is the non-spindle wave set.

Through the study of various detection algorithms and the analysis of a large number of experimental results, we found that the performance of most automatic spindle detection algorithms was not only different for different spindle datasets but also different for the same dataset. Most notably, different spindle wave detection algorithms, due to different settings, will detect a portion of non-coincident spindle waves in the same data. Based on this finding, we propose to first use two spindle detection algorithms to automatically detect spindles on the pre-processed sleep EEG data, after which the spindles detected by each algorithm were discriminated as true or false, with “1” representing *true spindles* and “0” representing *false spindles*, to facilitate the subsequent algorithm performance evaluation. Next, the spindles result set was fused, and the two data sets of overlapping and non-overlapping spindles were counted. Through the study of the overlapping spindle set, we found that 96.8% of the spindle set detected by the two algorithms together were true spindles, with such a high accuracy. As the overlapping spindle set were true spindles, we did not further process the overlapping spindles, which greatly reduced the workload, with no discernible effect on the results. A modified k-means clustering algorithm was used for the non-overlapping spindle dataset in this study, with the amplitude and frequency of non-overlapping spindles as variables for clustering. After that, the clusters, where a large number of non-spindles (i.e., spindles marked as 0) clustered, were removed. Next, the set of overlapping spindles and the set of clustered spindles were used as the final set of spindle results. Finally, the performance of the method applied in this study was compared with previously proposed methods.

## 3. Experimental results

After completing the spindle detection for 30 samples, we computed the recall, precision, specificity, accuracy and F1-score of the fusion algorithm, as well as their variance. We found that the performance of the fusion algorithm was satisfactory and stable. We extracted the spindle features from the spindle sample sets of the two populations (sleep-disordered and normal) and detected the spindles for feature analysis based on the improved algorithm presented herein. We studied the differences between sleep-disordered and normal controls primarily from two perspectives: (i) the density, amplitude, and frequency characteristics of C3 channel spindles in the central brain area as a whole (ii) the differences in the number of spindles between two groups of symmetrical channels, namely C3/C4 and F3/F4. We defined the density of spindle as the number of spindles that appear per minute during sleep, which is the number of spindles detected per sample divided by the duration of sleep (min). To facilitate the calculation, we used 8 h (480 min) of sleep EEG data processed uniformly from the first entry into the sleep stage. The spindle amplitude value is defined as the maximum peak-to-peak value of each spindle, which we first calculated using a band-pass filter of 11–16 Hz, followed by calculation of amplitude characteristics. For analyzing the frequency distribution of the spindles, we used the fast Fourier transform to perform a spectral analysis for the spindles and then derived the average frequency of each spindle. Next, we compared and analyzed the spindle characteristics between the sleep-disordered subjects and the normal subjects first as a whole and then among different individuals and drew conclusions.

Thirty subjects were scored on the Pittsburgh Sleep Quality Index before EEG acquisition in patients with sleep disorders. We analyzed the association between sleep quality scores and spindle features, which could reflect the impact of sleep disorders on spindle features. Table 1 shows the Pittsburgh Sleep Quality Index scores of 30 subjects in this paper. The higher the score, the worse the sleep quality, and those with a score greater than or equal to 11 were patients with sleep disorders, while the remaining were normal subjects.

**TABLE 1:** Pittsburgh Sleep Quality Index score.

Subject	Score	Subject	Score	Subject	Score
DS1	14	DS11	12	NS1	3
DS2	17	DS12	18	NS2	2
DS3	19	DS13	20	NS3	7
DS4	16	DS14	16	NS4	9
DS5	16	DS15	15	NS5	2
DS6	18	DS16	20	NS6	5
DS7	11	DS17	18	NS7	6
DS8	13	DS18	17	NS8	8
DS9	16	DS19	14	NS9	1
DS10	14	DS20	16	NS10	9

DS represents sleep-disordered subjects, NS represents normal subjects.

### 3.1. Analysis of sleep spindle density

**FIGURE 1:**
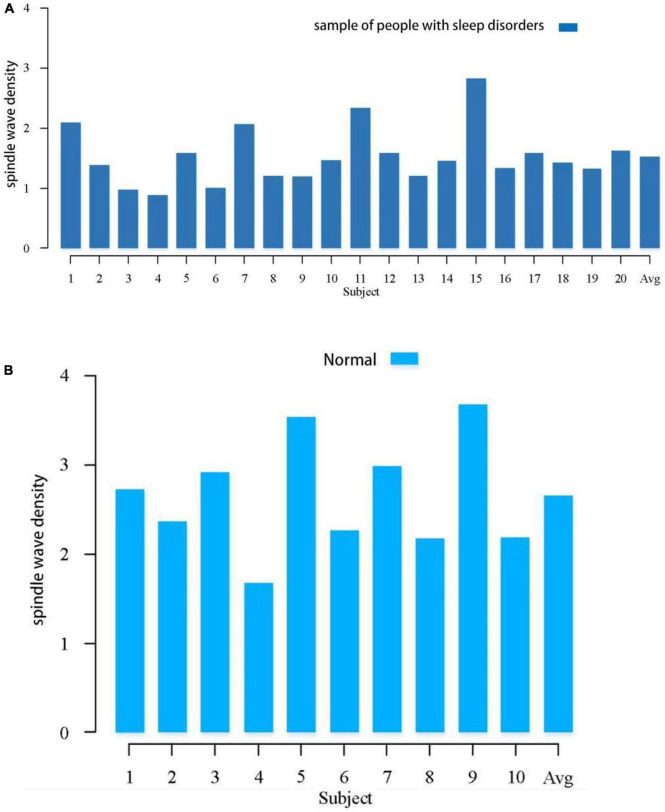
Comparison of spindle density between the sleep-disordered population and the normal population during the whole night sleep. **(A)** Spindle wave density in people with sleep disorders. **(B)** Spindle wave density in normal population.

Spindle density is a very important feature of spindles and has been shown to be closely related to individual development and some brain diseases. In this study, we first calculated the spindle density of each subject for overnight sleep. Figure 1 shows the comparison of spindle density between the sleep-disordered subjects and the normal subjects. In the sleep-disordered population, the highest spindle wave density was 2.83 waves per min, the lowest spindle wave density was only 0.89 waves per min, and the average spindle wave density was 1.53 waves per min. In contrast, the highest spindle wave density in the normal population could reach 3.5 waves per min, and the lowest was 1.68 waves per min. The average spindle wave density was 2.65 waves per min. The spindle density in the sleep disordered population was generally lower than that in the normal population, the *p*-value between them was 1.84 * 10^–8^ (*p* < 0.05 is significantly correlated), so the quality of sleep was positively correlated with the spindle density. Of course, there were individual sleep-disordered samples with higher spindle density than the normal population. For example, S1, S7, S11, and S15 had higher spindle densities than 2 per min. In summary, we can conclude that overall spindle wave density is correlated with the quality of sleep, the higher the spindle wave density, the better the quality of sleep, but it is also possible that the spindle wave density is not seriously affected by the low quality of sleep due to other reasons.

**FIGURE 2:**
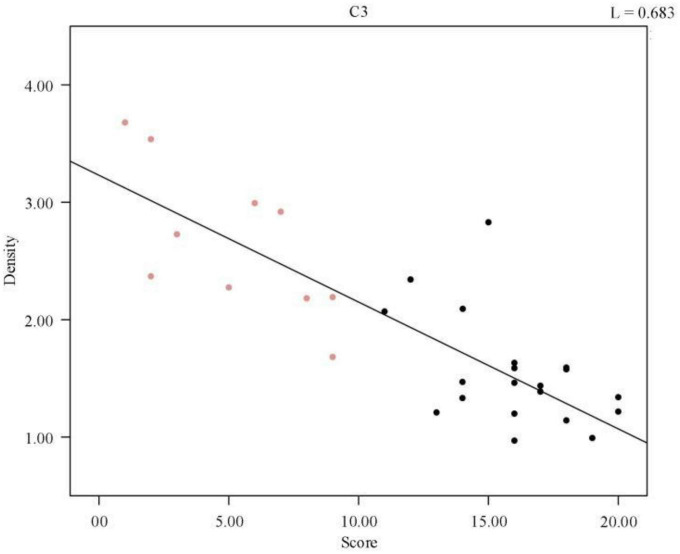
Regression line of individual spindle wave density and sleep quality score. The red data points represent the normal population data and the black data represent the sleep disorder population data.

To illustrate the relationship between sleep quality and spindle density more intuitively, a scatter plot of spindle wave density and sleep quality score was drawn and a regression line was fitted, as shown in Figure 2, the data points in red represent the normal population’s data, and those in black represent the sleep-disordered population’s data. It is evident from the figure that as the sleep quality score increased, the spindle density decreased. As the higher the sleep quality score, the worse the sleep quality, we concluded that the higher the spindle density, the better the sleep quality.

A complete sleep course consists of five to eight sleep cycles, with the start of one sleep cycle marked by entry into the non-rapid eye movement (NREM) period and the end marked by exit from the rapid eye movement (REM) period, which in turn constitutes a sleep cycle. The first cycle of sleep is of great interest because some sleep disorders are characterized by difficulty in falling asleep and easy wakefulness, so we analyzed the length of the first cycle of sleep in sleep-disordered subjects and normal subjects to identify any significant differences between them (*p*-value of 2.87 × 10^–8^, R^2^, L = 0.673). Table 2 presents the results of the duration of the first cycle of sleep and the number of spindles detected for the first cycle of sleep.

**TABLE 2:** Results of spindle wave number detection.

Subject	Time (min)	Spindles	Subject	Time (min)	Spindles
DS1	152.5	356	NS1	98.5	285
DS2	96.5	121	NS2	76.5	187
DS3	111.5	116	NS3	80.5	233
DS4	75.5	65	NS4	115	200
DS5	86	140	NS5	133.5	483
DS6	61	77	NS6	72.5	171
DS7	117	266	NS7	85.5	240
DS8	101.5	114	NS8	126	297
DS9	72	92	NS9	160	579
DS10	82	130	NS10	108.5	257
DS11	108.5	308	**\**	**\**	**\**
DS12	81	124	**\**	**\**	**\**
DS13	68.5	94	**\**	**\**	**\**
DS14	65	101	**\**	**\**	**\**
DS15	97	284	**\**	**\**	**\**
DS16	150	162	**\**	**\**	**\**
DS17	67	112	**\**	**\**	**\**
DS18	107	179	**\**	**\**	**\**
DS19	72.5	86	**\**	**\**	**\**
DS20	111.5	190	**\**	**\**	**\**
DS-Average	94.2	156.3	NS-Average	105.7	293.2

DS represents sleep-disordered subjects, NS represents normal subjects. DS - and NS - represent averages.

**FIGURE 3:**
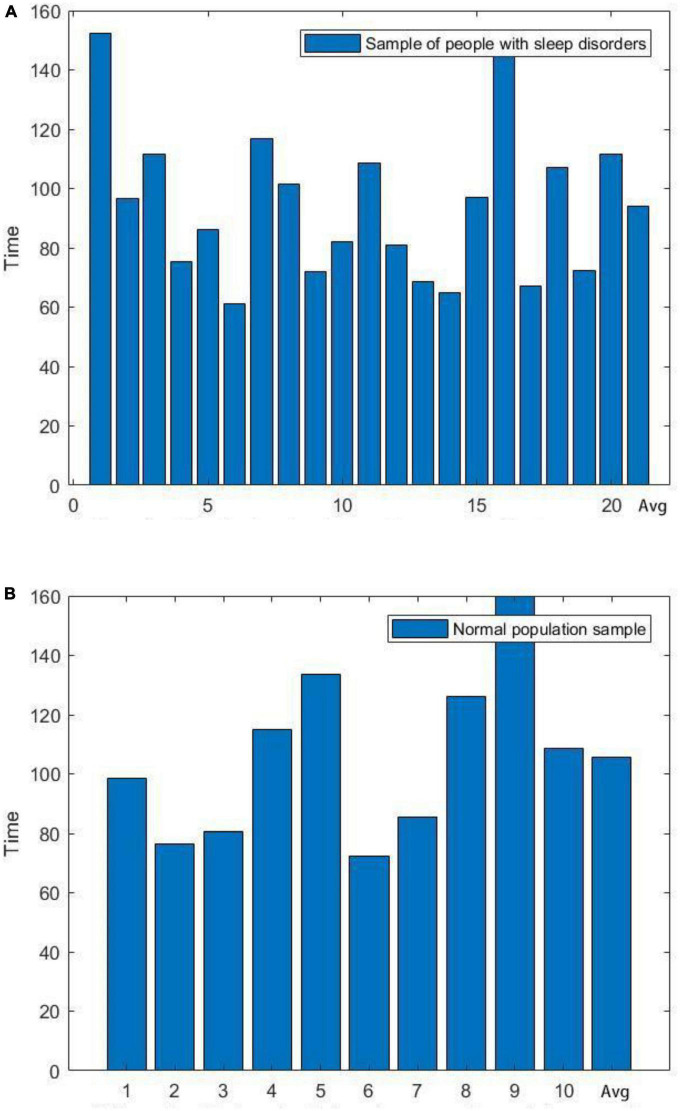
Comparison of the length of the first cycle of sleep between sleep-disordered and normal subjects. **(A)** Length of the first cycle of sleep for people with sleep disorders. **(B)** Length of the first cycle of sleep in a normal population sample.

Next, we plotted the histogram of the length of the first cycle of sleep for normal and sleep-disordered subjects, as depicted in Figure 3.

**FIGURE 4:**
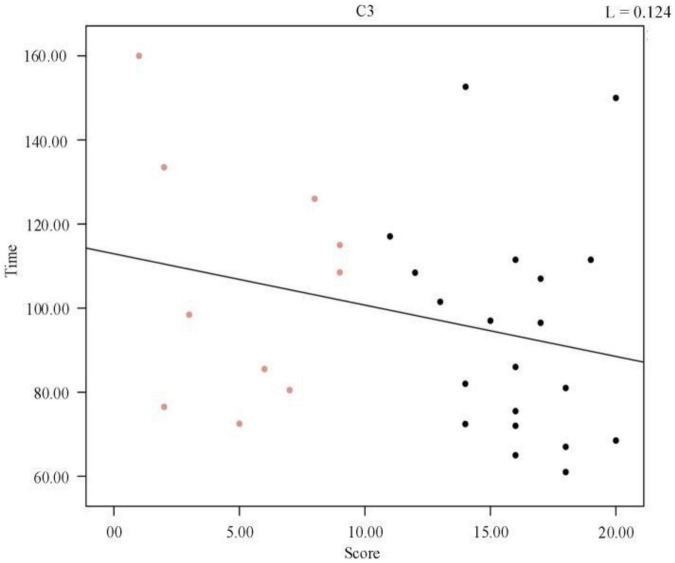
Regression line between the duration of the first cycle of individual sleep and sleep quality score.

We plotted the scatter plot between the duration of the first cycle of sleep and the sleep quality scores, as shown in Figure 4, and a regression line was fitted to the scatter plot, which verified that there was no significant relationship between the two. In the Pearson correlation analysis between sleep quality scores and mean frequency, the *p*-value was found to be 0.164 (R^2^, Linear, L = 0.124), suggesting that the correlation was not significant. We concluded that no significant correlation existed between the duration of the first cycle of sleep and the sleep quality scores, i.e., the quality of sleep was not related to the duration of the first cycle of sleep.

**FIGURE 5:**
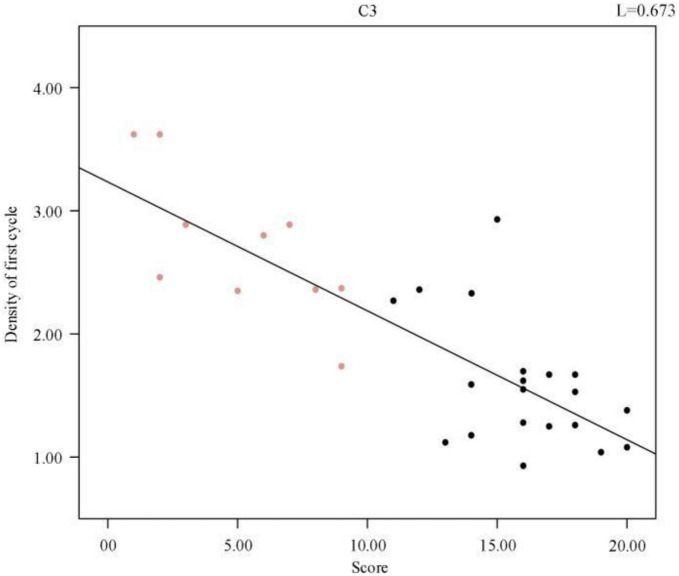
Regression line between individual sleep first cycle spindle density and sleep quality score.

We further analyzed a correlation between the first cycle of spindle density and sleep quality score and obtained a *p*-value of 2.87 × 10^–8^. Figure 5 shows the scatter plot of spindle density and sleep quality score in the first cycle of sleep. Clearly, the spindle density in the first cycle of sleep also had a significant negative correlation with the sleep quality score, and as the spindle density increased, the sleep quality score continuously decreased. In other words, the higher the spindle density, the better the sleep quality.

### 3.2. Comparative analysis of sleep spindle wave frequencies

**FIGURE 6:**
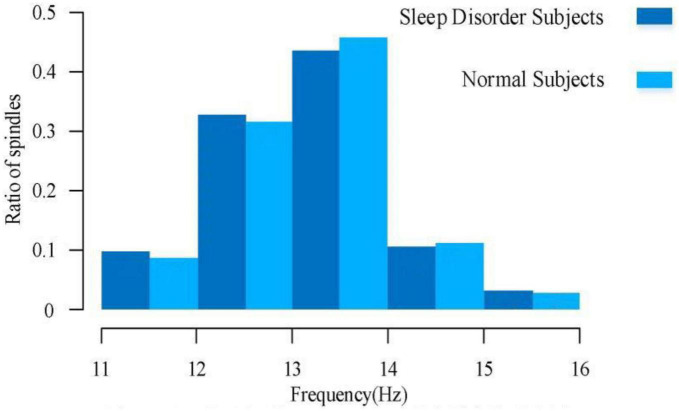
Overall spindle frequency distribution of sleep-disordered and normal subjects.

For a comparative analysis of frequency, it can be learned from the existing low-density EEG studies that the spindle frequencies are mainly distributed between 11.0–16.0 Hz, with a large number of spindle frequencies distributed between 12.0–14.0 Hz, of which 11.0–12.5 Hz spindles are mostly found in the prefrontal lobe of the brain, and 12.5–14.0 Hz spindles are mostly found in the central brain area. There are fewer spindles at low and high frequencies, but irregular spindles are not excluded for various reasons. Figure 6 illustrates the spindle frequency distribution of sleep-disordered subjects and normal subjects as a whole. We divided the frequency distribution of the spindle into five parts, and the overall distribution of frequency was basically consistent with previous studies. We found no significant difference in the spindle frequency distribution between 12–14 Hz and 77.2% in the normal population, which was 76.4% of the total sleep-disordered population. For the 11–12 Hz range, the percentage of spindles was slightly higher in the sleep-disordered population than in the normal population by 1.1%. In the range 14–15 Hz, the proportion of spindles was slightly higher in the normal population than in the sleep-disordered population by 0.6%. Finally, in the range 15–16 Hz, the proportion of spindles was slightly higher in the sleep-disordered population than in the normal population by 0.3%. Through the analysis of these data, we initially determined that the distribution of spindle frequencies did not differ significantly in the C3 channel of the central brain region. However, since we only used the C3 channel for spindle frequency analysis, we could not fully conclude that the sleep disorders did not have any effect on the spindle frequencies in the whole brain. This result can only indicate that there was no significant difference in the distribution of spindle frequencies between the sleep-disordered population and the normal population in the C3 channel.

**FIGURE 7:**
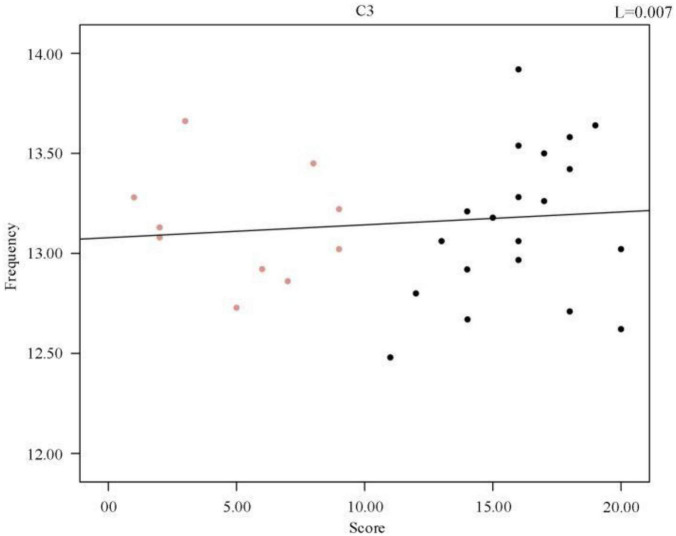
Regression line of individual spindles’ mean frequency and sleep quality score.

To further investigate the relationship between frequency and sleep quality, a Pearson correlation analysis was performed between the subjects’ sleep quality scores and the mean spindle wave frequency of each sample, *p* = 0.667 (R^2^, Linear, L = 0.007). A scatter plot between the mean spindle wave frequency and sleep quality scores of individuals was then plotted and a regression line was fitted, which revealed that there was no correlation between sleep quality scores and mean frequency. As shown in Figure 7, there was no correlation between the change in mean frequency and the change in sleep score. Therefore, we synthesized the data analysis overall and between individuals. The average frequency of spindles in the C3 channel did not change significantly in both sleep-disordered and normal subjects.

### 3.3. Comparative analysis of sleep spindle amplitude

**FIGURE 8:**
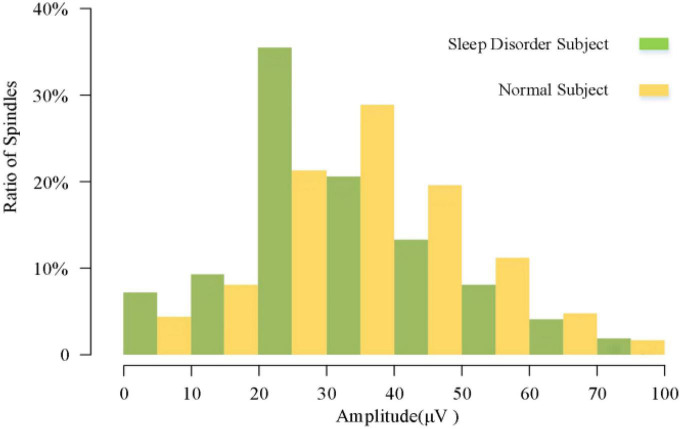
Overall spindle amplitude distribution of sleep-disordered subjects and normal subjects.

In the analysis of spindle amplitude value characteristics, we divided the spindle amplitude value into eight intervals, mainly distributed between 0 and 60 μV, and only a few spindles appeared above 60 μV. It can be seen from Figure 8 that the amplitude of the spindle was mostly distributed between 20 and 40 μV in both the sleep-disordered and normal subjects, with 56.1% of the spindles distributed between 20 and 40 μV in the sleep-disordered population samples and 50.3% of the spindles distributed between 20 and 40 μV in the normal population samples. Between 20 and 30 μV, the proportion of spindles was 14.2% higher in the sleep-disordered population than in the normal population. However, between 30 and 40 μV, the proportion of spindles was 8.3% higher in the normal population than in the sleep-disordered population. Furthermore, between 40 and 60 μV, the proportion of spindles in the normal population was 9.4% higher than that in the sleep-disordered population. Overall, the percentage of spindles in the sleep-disordered population was higher in the 0–30 μV range than in the normal population, and the percentage of spindles above 30 μV was higher in the normal population than in the sleep-disordered population. We found from the above analysis that the spindle amplitude of the normal population was higher than that of the sleep-disordered population on average. We performed a Pearson correlation analysis between the sleep quality scores of the subjects and the mean spindle amplitude of each sample. The *p*-value was 1.33 × 10^–4^ (R^2^, Linear, L = 0.412), which reflected a significant correlation between the two variables.

**FIGURE 9:**
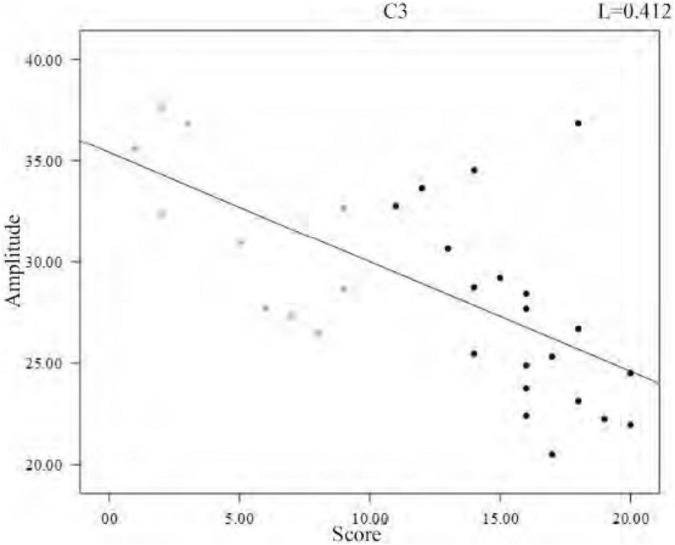
Regression line of individual spindles mean amplitude and sleep quality score.

To further investigate the relationship between amplitude and sleep quality, we then plotted the scatter plot between the mean amplitude of the spindle of the individuals and the sleep quality scores with a fitted regression line, and it can be clearly seen from Figure 9 that the mean amplitude of the spindle decreased as the score increased. Altogether, from the overall distribution of amplitudes and the variability between individuals, we can conclude that the average amplitude of spindle was lower in the sleep-disordered population.

### 3.4. Analysis of the number of spindles in symmetric channels

In several previous studies, the EEG signals of the left and right hemispheres mostly showed symmetry in amplitude and frequency, and the symmetrical channels showed the same characteristic changes in response to external stimuli. In our further analysis, we performed a comparative analysis of the number of spindles in symmetrical channels to investigate whether sleep disorder disorders have an impact on the symmetry of the number of spindles.

**FIGURE 10:**
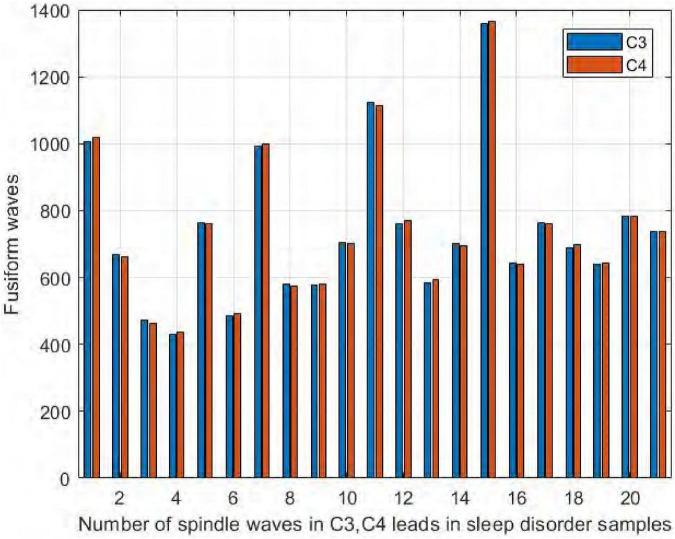
Comparison of the number of spindles in C3 and C4 channels in the sleep-disordered population.

We collected EEG signals from six channels symmetrical to the central brain region, frontal lobe, and occipital lobe. We used four channels, C3, C4, F3, and F4, to study the difference in spindle performance on the left and right hemispheres. Figure 10 shows the comparison of the number of spindles in C3 and C4 channels in the central brain region on the sleep-disordered as well as the normal subjects. As shown in Figure 10, in all 20 samples of the sleep disorder population, the difference in the number of spindles ranged from ±13. In sample DS1, the number of spindle waves in channel C4 was 13 more than that in channel C3, and in sample DS19, the number of spindles in channel C4 was only 1 more than that in channel C3. So it can be said that there was no variability in the number of spindle waves in the two symmetrical channels C3/C4.

**FIGURE 11:**
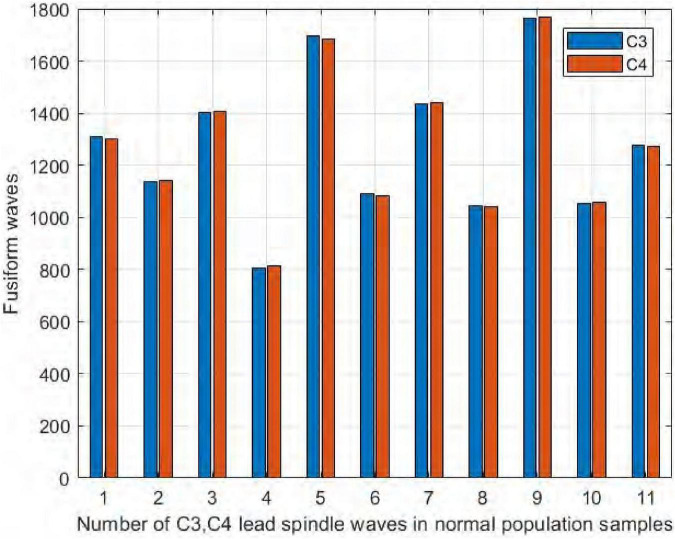
Comparison of the number of spindles in C3 and C4 channels in normal subjects.

Similarly, it can be seen in Figure 11 that the number of spindles in the normal subjects was not significantly different in C3/C4, with a difference range of ±12. The largest difference was in NS5, where C3 had 12 more spindles than C4, and in NS9, where C4 had only 3 more spindles than C3. We thus concluded that normal and sleep-disordered subjects did not show differences in the feature of the number of spindles in the symmetric channels C3/C4.

**FIGURE 12:**
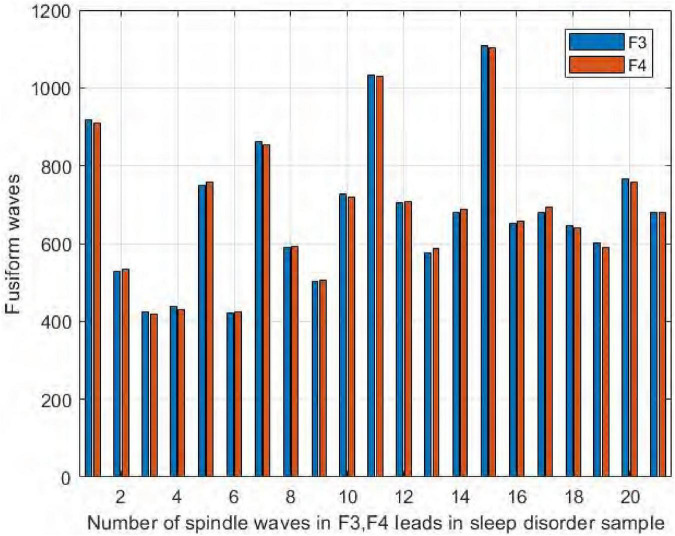
Comparison of the number of spindles in channels F3 and F4 in the sleep-disordered subjects.

We also compared the number of spindles in the channels F3 and F4 of the frontal brain region. As seen in Figure 12, we found no significant difference in the number of spindles in symmetric channels F3 and F4 in the sleep-disordered subjects. In all 20 subjects, the difference in the number of detected spindles between the two channels was always no more than 12. In sample DS17, the number of spindles in channel F4 was 12 more than that in channel F3, and in samples NS6 and NS12, the difference was only 3. As with C3 and C4, the number of spindles in F3 and F4 was almost identical in the sleep-disordered subjects, with no significant differences.

**FIGURE 13:**
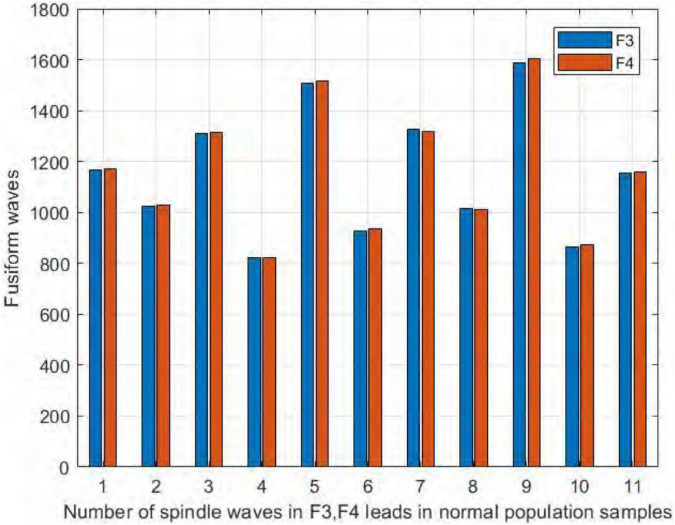
Comparison of the number of spindles in channels F3 and F4 in normal subjects.

Similarly, it can be seen from Figure 13 that the number of spindles in the normal subjects was not significantly different in F3/F4, with the largest difference in NS5, where F3 had 15 more spindles than F4, and in NS9, where F4 had only 3 more spindles than F3.

### 3.5. Comparison of results

**FIGURE 14:**
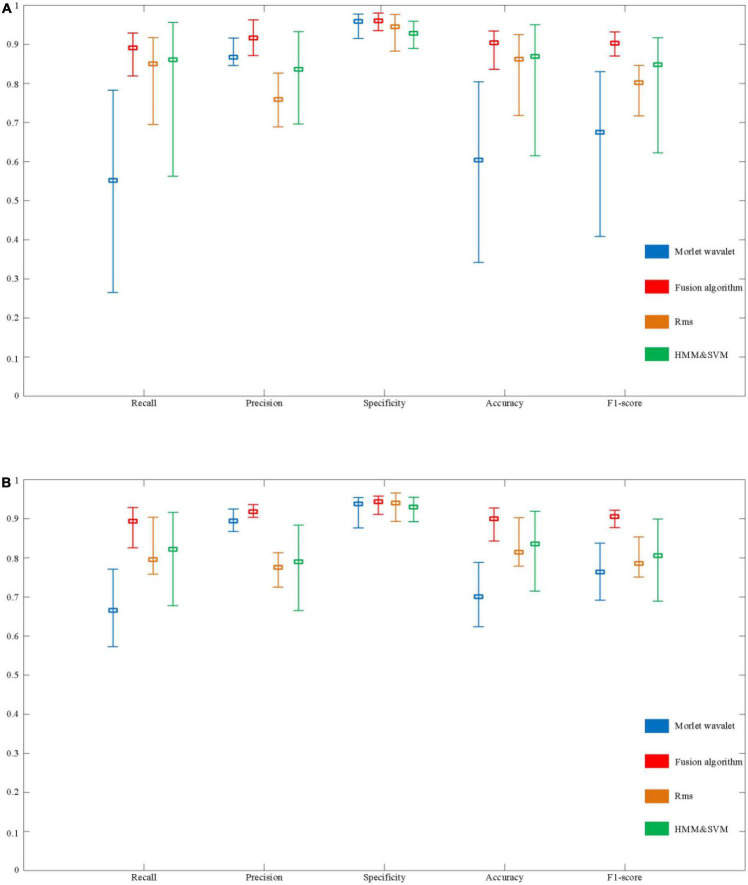
Evaluation of the performance of automatic spindle wave detection based on fusion algorithm. **(A)** Performance comparison of detection methods based on sleep disorder data. **(B)** Performance comparison of detection algorithms based on normal population data.

To illustrate the effectiveness of the method proposed in this paper, we compared the algorithm proposed in this paper with Morlet wavelets, RMS and HMM & SVM. Figure 14 shows the performance of the fusion algorithm compared with the other three algorithms.

In a sample of people with sleep disorders, the Recall of the fusion algorithm can reach a maximum of 92.9% and a minimum of 81.9%, with a mean value of 89.1%. The highest, lowest, and average Recall in the normal population can reach 92.9, 82.6, and 89.3%, respectively. Although there is still a difference of about 10% between the highest and lowest, the overall recall rate has improved significantly compared to the above three detection algorithms. In terms of Precision, the maximum value for the sleep disorder sample is 96.3%, the minimum value is 87.1%, and the mean value is 91.6%. In the normal sample, it was 93.6, 90.4, and 91.8%, respectively. It can be seen that the average Precision of the fusion algorithm is significantly better than the RMS and HMM & SVM algorithms, and slightly better than the Morlet wavelet algorithm. Besides, specificity performs equally well. Finally, as shown in Figure 14, the performance on Accuracy and F1-score is also better than the other three algorithms, with mean values of 90.4 and 90.3% in the sleep disorder data, respectively.

As shown in Figure 14, it can be seen that the maximum, minimum, and average values of each evaluation index of the fusion algorithm are significantly improved compared with the other three algorithms. In order to further verify the stability of the performance of the fusion algorithm in 30 samples (including sleep disorder samples and normal samples), the variance of each performance index of the four algorithms was calculated, as shown in Table 3. The variance of the five evaluation metrics of the fusion algorithm are significantly lower than those of the other three algorithms, indicating that the fusion algorithm proposed in this paper has stronger stability in different samples.

**TABLE 3:** Four automatic detection algorithms to evaluate metric variance.

Evaluation metrics	Recall	Precision	Specificity	Accuracy	F1-score
RMS	28.55	13.13	5.43	22.85	10.36
Morlet wavelet	165.74	4.55	4.56	138.16	114.40
HMM & SVM	80.19	42.89	2.39	59.09	48.63
Fusion algorithm	8.84	3.06	2.89	6.50	2.49

## 4. Discussion

In this study, we proposed a new spindle wave detection algorithm. Our proposed algorithm has a large performance improvement compared to other algorithms. Based on the proposed algorithm, we analyzed the differences in the density, frequency, amplitude, and symmetry of the spindles between 20 sleep-disordered subjects and 10 normal subjects. We found that the two characteristics of spindle density and amplitude were significantly different between sleep-disordered and normal populations. The higher the spindle density, the better the quality of sleep. Similarly, the higher the amplitude of the spindle, the better the sleep quality. We found no differences in the frequency of the spindles between the sleep-disordered and normal subjects in terms of the number of spindles in symmetrical channels C3/C4 and F3/F4.

Neuropsychiatric disorders are typically characterized by the phenomenon of wakefulness, but sleep disturbance is a prominent feature. Although sleep deprivation is usually considered secondary, it can induce psychosis and trigger or aggravate a range of disorders. Treating sleep disorders can improve symptoms of neuropsychiatric disorders and enhance cognitive abilities (Manoach and Stickgold, 2009). This suggests that abnormal sleep is not just an accidental phenomenon but may also lead directly to neuropsychiatric disorders.

Our results illustrate the relationship between sleep fusiform waves and sleep quality, which is similar to the results of previous studies (Vallat et al., 2017). But there are also many differences. The context of our study was different in that they were studying sleep quality in different stressful situations and none of these subjects had sleep disorders. In addition to that, our subjects were between 20–40 years old, they were from different places and in different occupations, which makes our results more generalizable.

In conclusion, despite the long-standing association of various forms of sleep disorders with schizophrenia, it is difficult to determine the exact administration of the problem and its relationship with pathophysiology, cognitive deficits, and symptoms. It has been shown that in healthy young adults, reduced spindle density is associated with elevated scores on the Psychotic Disposition Scale and elevated thalamic glutamine and glutamate levels, further supporting a mechanical link between spindles, enhanced thalamic excitation, and the risk of psychosis (Lustenberger et al., 2015). Meanwhile, spindle defects have also been associated with poorer executive performance, memory, and a lower Intelligence Quotient (IQ) (Fogel and Smith, 2011; Manoach et al., 2014; Schilling et al., 2016), suggesting a more general role for the spindle in cognitive deficits. To identify therapeutic targets, future work must define sleep abnormalities in different disorders and better study the associated characterization of spindles to better address issues related to these disorders.

In a previous study, Manoach et al. evaluated sleep abnormalities in adults with a recent diagnosis of psychosis, non-psychotic first-degree relatives of patients with schizophrenia, and two samples of healthy controls matched to the patients and their relatives (Manoach et al., 2014). Patients with early course of diagnosed schizophrenia had significantly lower spindle range NREM sleep EEG power, spindle amplitude, and spindle density compared with healthy controls and other psychiatric patients. Spindle activity was also reduced in relatives of patients with schizophrenia compared with controls. Willoughby et al. used a large cross-sectional sample of adolescents and calculated sigma power, spindle characteristics, and cognitive data for fast (∼13 Hz), central, and slow (∼11 Hz) frontal sleep spindles. Their results showed lower absolute sigma power at older ages (late pubertal development), faster spindle density, shorter spindle duration, smaller amplitude, and faster mean frequency than at younger ages. Spindle characteristics are not directly related to cognition. According to previous investigations, peak sigma frequency increases in childhood and adolescence (Tarokh and Carskadon, 2010), spindle duration and amplitude increase in early childhood (McClain et al., 2016), and spindle density is greater in older adolescents compared with that in younger adolescents (Bodizs et al., 2014). Recently, longitudinal changes in peak sigma frequency and sigma power during adolescence were observed (Campbell and Feinberg, 2016). Throughout childhood, the spindle duration gradually decreases with age, peaks at adolescence and then declines throughout adulthood (Purcell et al., 2017). There are significant differences in the spindle waves produced by different individuals during sleep, and these differences are reflected in the characteristics of spindle wave density, amplitude, duration, and frequency. According to current research, factors that cause changes in these characteristics of spindle waves include age, gender, intelligence, and related diseases (Ujma et al., 2016; Weng et al., 2020). Kiesow and Surwillo (1987) analyzed the differences between children with Attention Deficit and Hyperactivity Disorder (ADHD) and healthy children using classical spindle parameters and found no significant differences in the number, spindle density, amplitude, and frequency peaks of spindles per sleep stage owing to the lack of classical parameters.

The main limitation of this study is that we collected EEG data from only 30 subjects and processed them uniformly for 8 h, and then extracted the spindle wave features. The small amount of subject may result in less accurate performance of some of their characteristics, where differences between the two groups of sleep-disordered and normal populations may require larger sample sizes to make the results more generalizable. We cannot yet fully and directly consider other factors that have been shown to influence the spindles. Besides, because we only used C3 channel for spindle wave frequency analysis, we cannot state that sleep disorder disease does not have any effect on spindle wave frequency, and we need to further analyze the relationship between spindle wave frequency and sleep quality in other parts of EEG signal.

In summary, our proposed detection method can effectively improve the accuracy of sleep spindle wave detection with stable performance. Meanwhile, our study shows that the spindle density, frequency and amplitude are different between the sleep-disordered and normal populations. If true, these characteristics can be used as indicators of the extent to which the disease has been developed and can represent reliable objective parameters. Early intervention through sound and electrical stimulation as well as pharmacological treatment is also possible (Marshall et al., 2006; Mednick et al., 2013; Magalini and Masiero, 2015). Therefore, future studies should assess whether the measurement of these characteristics is valid and then improve the characteristic values of sleep spindles through the use of timely interventions. This work will eventually prepare for early therapeutic interventions based on the physiology of sleep disorders. The characteristic contrast of sleep spindles can be used as a prognostic biomarkers, which are widely used to identify the likelihood of a clinical event, disease recurrence or progression in patients. For example, lower spindle parameters at initial assessment may be associated with a poorer clinical prognosis, while higher spindle parameters may lead to a better clinical outcome. Future studies should examine the relationship between having a psychiatric disorder, like Parkinson’s disease, schizophrenia, autism, insomnia, and Alzheimer’s disease, and the sleep spindles features, and improve its poor effects by enhancing or manipulating the sleep spindles.

In the subsequent work, more aspects can be explored to ascertain the differences of spindle characteristics between the two groups of people, such as the differences in spindle duration or in the average amplitude of spindle in symmetrical channels. A more comprehensive feature analysis can provide a more accurate objective basis for the clinical diagnosis of sleep disorders. Besides, in this experiment, patients with insomnia disorder and sleep apnea disorder were selected. However, the classification of sleep disorders also includes sleep wakefulness circadian disorder, narcolepsy disorder, etc. In future studies, it is necessary to continue to study the changes of spindle wave characteristics in patients suffering from other types of sleep disorders diseases.

## Data availability statement

The original contributions presented in this study are included in the article/supplementary material, further inquiries can be directed to the corresponding authors upon reasonable request, and according the policies of Tianjin University, Tianjin University of Technology, Tianjin Medical University General Hospital, Xuanwu Hospital, Capital Medical University.

## Ethics statement

The studies involving human participants were reviewed and approved by Xuanwu Hospital, Capital Medical University. The patients/participants provided their written informed consent to participate in this study.

## Author contributions

CC, KW, and ZH recorded the original experiment data, analyzed the experiment data, and penned the manuscript. CC, LL, DM, and AB wrote parts of the manuscript. WY and JL designed the experiment and revised the manuscript. All authors contributed to the article and approved the submitted version.
